# The STOP1-MATE-Citrate Axis Confers Inorganic Sn Tolerance in Plants

**DOI:** 10.3390/plants15142231

**Published:** 2026-07-22

**Authors:** Zixuan Zhao, Ting He, Yi Fan, Hongxia Chang, Zhixuan Du, Longfei Zhu, Guanping Feng

**Affiliations:** Key Laboratory of Jiangxi Province for Biological Invasion and Biosecurity, School of Life Sciences, Jinggangshan University, Ji’an 343009, China

**Keywords:** inorganic Sn, phytotoxicity, STOP1, MATE, citrate

## Abstract

Inorganic tin (Sn) is an emerging pollutant whose phytotoxicity and plant detoxification mechanisms remain poorly understood. Here, we demonstrate that inorganic Sn severely inhibits root growth and triggers ROS accumulation in *Lemna minor* and Arabidopsis. We identified the transcription factor STOP1 as a critical regulator of Sn tolerance. Mechanistically, STOP1 confers Sn tolerance primarily by activating the citrate transporter MATE, contrasting sharply with Al tolerance which relies heavily on the malate transporter *ALMT1*. Inorganic Sn stress strongly induced root citrate exudation, and the *mate* mutant displayed severe Sn hypersensitivity. Exogenous citrate application effectively rescued root growth by impeding Sn uptake and attenuating ROS overaccumulation. Our study reveals that the STOP1-MATE-citrate axis constitutes a pivotal detoxification mechanism against inorganic Sn, providing novel insights into how plants adapt to heavy metal stress through distinct organic acid secretion pathways.

## 1. Introduction

Tin has been utilized by humans since the Bronze Age, with its alloys historically fashioned into domestic items such as tableware and drinking vessels. The Industrial Revolution marked a shift toward the deliberate production of inorganic tin compounds for diverse applications, followed by the emergence of organotin manufacturing by the 1940s [1]. Today, inorganic tin compounds—where the tin atom exists primarily in divalent or tetravalent states—hold immense technical and economic value [2]. Industrially relevant examples are ubiquitous: tin (II) chloride and tin (II) sulfate are widely used for steel plating; tin (II) fluoride serves as an anti-caries ingredient in toothpastes; tin (IV) oxide is combined with pigments as ceramic colorants; and tin (IV) chloride acts as a precursor in organotin synthesis and is utilized in military applications such as smoke bombs [3]. This widespread industrial application has deeply integrated tin chemistry into modern human life.

Historically, metallic Sn has been considered virtually non-toxic, as ingested Sn is poorly absorbed by the body, and naturally occurring Sn toxicity in plants, animals, or humans has not been documented under baseline environmental conditions [4]. Typically, natural concentrations of inorganic Sn in air, soil, and water are minimal, with the natural background value of Sn in soil being approximately 2.6 mg kg^−1^ [5]. However, this baseline shifts drastically due to anthropogenic activities. Mining, sewage irrigation, atmospheric deposition, and the use of industrial chemicals like PVC stabilizers have led to severe Sn enrichment in local environments. For instance, a spatial survey in the Yangtze River Delta revealed an average soil Sn content of 8.35 mg kg^−1^—far exceeding the national background value—with over 90% of the surveyed area showing enrichment characteristics [6]. In aqueous solutions near tin processing industries, inorganic Sn levels severely exceed safe limits. Such extreme enrichment poses a significant threat to local ecosystems, and the detrimental impacts on immobile plants, in particular, should not be overlooked.

Despite escalating environmental tin pollution, few ecotoxicological studies on inorganic Sn compounds have been published, and its toxicity is generally considered low [7]. Nevertheless, emerging evidence challenges this assumption [8]. One study demonstrated that both tin (II) and tin (IV) salts can inhibit the growth of *planktonic cyanobacteria*, with toxicity escalating under higher tin concentrations, elevated pH levels, and prolonged exposure times [9]. Given that plants are rooted in their environment and cannot evade heavy metal stress, the potential for inorganic Sn to exert phytotoxicity is substantial [10]. Yet, how inorganic Sn affects higher plant growth, and whether plants possess inherent detoxification mechanisms to cope with this emerging contaminant, remains largely unknown.

The massive discharge of inorganic Sn into the environment poses an increasingly severe threat to plants growing in aquatic and soil ecosystems. Addressing this pressing issue, we investigated the inhibitory effects of inorganic Sn stress on plant growth—particularly root growth—using the aquatic plant *Lemna minor* and the model plant Arabidopsis. We discovered that inorganic Sn severely inhibits root growth and triggers reactive oxygen species (ROS) accumulation. Furthermore, we uncovered that plants can counter inorganic Sn stress by secreting citrate via the STOP1-MATE pathway. Our findings elucidate a pivotal detoxification mechanism against inorganic Sn, providing novel insights into how plants adapt to heavy metal stress through distinct organic acid secretion pathways.

## 2. Results

### 2.1. Inorganic Sn Stress Inhibits Lemna Minor Root Growth

**Figure 1:**
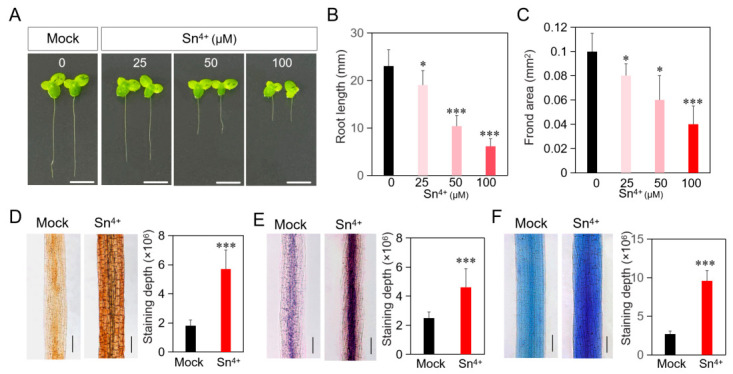
Inorganic Sn inhibits *Lemna minor* growth. (**A**) Phenotypes of inorganic Sn-induced growth inhibition in *Lemna minor*. Scale bar = 2 mm. (**B**) The root length of plants in (**A**). Data are presented as the mean ± SE (*n* > 10). (**C**) The area of fronds in (**A**). Data are presented as the mean ± SE (*n* > 10). (**D**) DAB staining of H_2_O_2_ and (**E**) NBT staining of O_2_^−^ and (**F**) Trypan blue staining in roots treated with 50 µM SnCl_4_ for 1 h. Data are presented as the mean ± SE (*n* > 5). Asterisks indicate statistically significant differences (***, *p* < 0.01; *, *p* < 0.05).

As one of the smallest flowering aquatic plants, *Lemna minor* is highly sensitive to environmental changes and is widely used as a bioindicator for monitoring water quality. By adding different concentrations of SnCl_4_ to Hoagland’s solution for *Lemna minor* cultivation, we found that SnCl_4_ at concentrations as low as 25 μM inhibited *Lemna minor* root growth. At 50 μM, the inhibitory effect was highly significant, and the frond area also decreased significantly (Figure 1A–C). This contrasts sharply with previous reports suggesting the low ecotoxicity of inorganic Sn. DAB and NBT staining revealed that SnCl_4_ induced excessive accumulation of hydrogen peroxide (H_2_O_2_) and superoxide anion (O_2_^−^) in *Lemna minor* roots, indicating that inorganic Sn stress triggers massive ROS generation, thereby affecting *Lemna minor* growth (Figure 1D,E). Furthermore, Trypan blue staining demonstrated that SnCl_4_ treatment resulted in a substantial increase in cell death in the roots, further confirming that inorganic Sn stress exerts severe adverse effects on *Lemna minor* growth (Figure 1F).

### 2.2. Transcription Factor STOP1 Participates in Plant Resistance to Sn Stress

**Figure 2:**
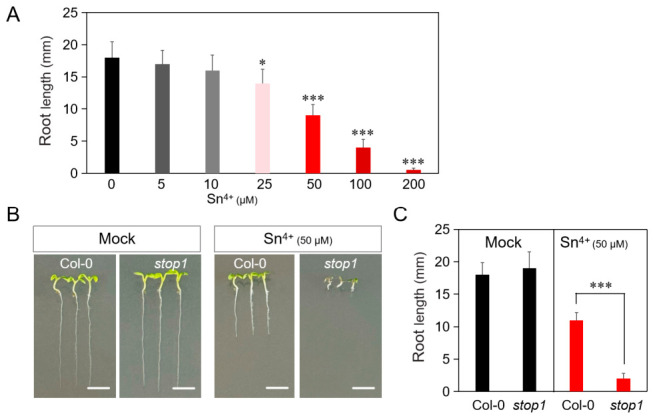
Mutation in STOP1 leads to hypersensitivity to Sn. (**A**) Dose–response effect of Sn on root growth inhibition. Data are presented as the mean ± SE (*n* > 10). Asterisks indicate statistically significant differences (***, *p* < 0.01; *, *p* < 0.05). (**B**) Phenotypes of wild-type (Col-0) and *stop1* mutant Arabidopsis plants grown on 1/2MS medium with or without (Mock) 50 µM SnCl_4_. Scale bar = 3 mm. (**C**) The root length of plants in (**B**). Data are presented as the mean ± SE (*n* > 10). Asterisks indicate statistically significant differences (***, *p* < 0.01).

To investigate the underlying mechanism by which inorganic Sn inhibits root growth, we first added SnCl_4_ to 1/2 MS medium and found that 50 μM SnCl_4_ significantly inhibited root growth in the model plant Arabidopsis, which is consistent with the observations in *Lemna minor* (Figure 2A). Subsequently, we extensively screened Arabidopsis mutants on 1/2 MS medium containing 50 μM SnCl_4_. We found that *stop1*, a loss-of-function mutant of the key aluminum-tolerance transcription factor STOP1, was hypersensitive to Sn (Figure 2B,C). The experimental result clearly demonstrates that the transcription factor STOP1 plays a positive regulatory role in plant resistance to inorganic Sn.

### 2.3. The Transcription Factor STOP1 Confers Sn Tolerance Primarily via the MATE

**Figure 3:**
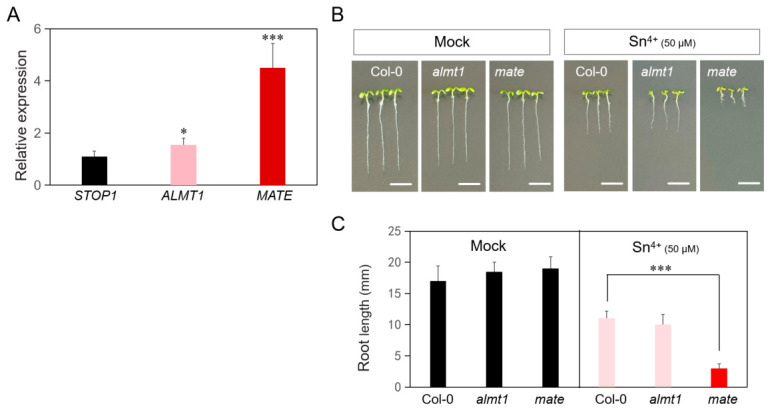
STOP1-mediated Sn tolerance relies on the *MATE*. (**A**) qPCR analysis of genes expression in the plants after 2 h of 50 µM Sn treatment. The data show one of three independent experiments. (**B**) Phenotypes of wild-type (Col-0), *almt1* and *mate* mutant Arabidopsis plants grown on 1/2MS medium with or without (Mock) 50 µM SnCl_4_. Scale bar = 3 mm. (**C**) The root length of plants in B. Data are presented as the mean ± SE (*n* > 10). Asterisks indicate statistically significant differences (***, *p* < 0.01; *, *p* < 0.05).

As a prominent transcription factor in plant aluminum resistance, the mechanism of action of STOP1 has been thoroughly elucidated. It can directly bind to the promoters of the genes encoding the malate transporter ALMT1 and the citrate transporter MATE, regulating the secretion of these two organic acids [11]. This subsequently facilitates the chelation of aluminum ions by these organic acids, effectively mitigating aluminum toxicity. Does STOP1 employ the same mechanism in its involvement in resistance to inorganic Sn stress? To address this question, we first used quantitative real-time PCR (qPCR) to examine the expression levels of *STOP1* and its target genes *ALMT1* and *MATE* under SnCl_4_ treatment. We found that the target genes *ALMT1* and *MATE* could be induced, with the increase in *MATE* expression being particularly prominent (Figure 3A). Subsequently, we analyzed the sensitivity of *almt1* and *mate* loss-of-function mutants to SnCl_4_. Compared to the wild-type control Col-0, the *almt1* mutant exhibited almost no difference in sensitivity, whereas the *mate* mutant displayed a severely hypersensitive phenotype (Figure 3B,C). These findings indicate that STOP1 confers inorganic Sn tolerance mainly via MATE-dependent citrate secretion, which stands in stark contrast to aluminum tolerance in Arabidopsis that primarily depends on ALMT1-mediated malate secretion.

### 2.4. Inorganic Sn Stress Induces Citrate Exudation

**Figure 4:**
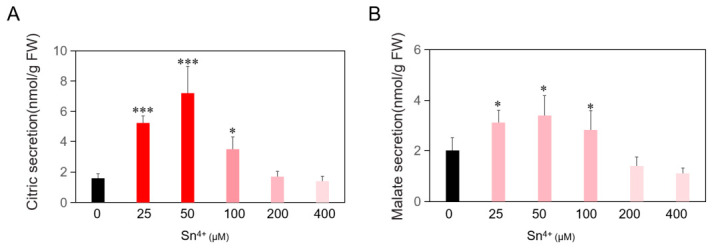
Sn elevates citrate exudation dramatically. (**A**) HPLC-MS/MS analysis of citrate exuded from Arabidopsis roots following 24 h exposure to 50 µM SnCl_4_. (**B**) HPLC-MS/MS analysis of malate exuded from Arabidopsis roots following 24 h exposure to 50 µM SnCl_4_. Three biological replicates were used, and significance was assessed via Student’s *t*-test (***, *p* < 0.01; *, *p* < 0.05).

To better elucidate the role of citrate exudation in inorganic Sn stress tolerance, we quantified the root exudates of Arabidopsis wild-type Col-0 under SnCl_4_ treatment using HPLC-MS/MS. Notably, 25 μM SnCl_4_ dramatically elevated citrate levels to more than 4-fold of the control, whereas malate accumulation showed a much more modest increase, peaking at approximately 1.7-fold under 50 μM SnCl_4_ treatment (Figure 4). These findings demonstrate that inorganic Sn stress triggers the exudation of both malate and citrate from Arabidopsis roots, with the induction of citrate being remarkably more pronounced. Collectively, this suggests that plants actively augment organic acid exudation to mitigate inorganic Sn stress, relying predominantly on citrate.

### 2.5. Exogenous Citrate Significantly Alleviates Sn Stress

**Figure 5:**
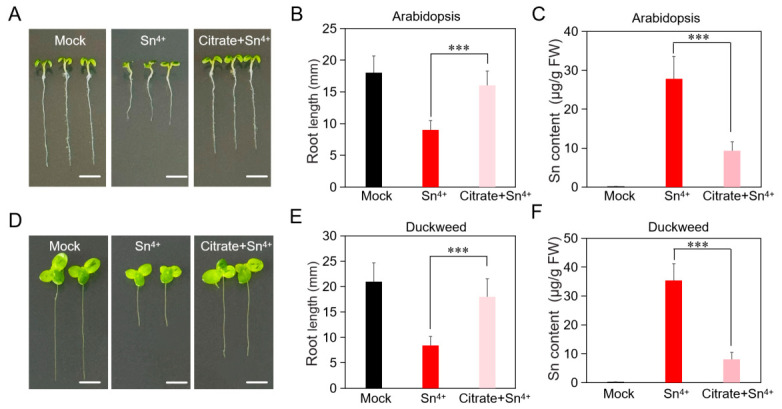
Exogenous application of citrate alleviated Sn stress. (**A**) Phenotypes of wild-type Arabidopsis seedlings grown on 1/2 MS medium under the treatments: Mock, 50 µM Sn, 50 µM Sn and 10 µM citrate. Scale bar = 3 mm. (**B**) The root length of plants in (**A**). Data are presented as the mean ± SE (*n* > 10). Asterisks indicate statistically significant differences (***, *p* < 0.01); (**C**) The Sn content of plants in (**A**). Three biological replicates were used, and significance was assessed via Student’s *t*-test (***, *p* < 0.01). (**D**–**F**) show the analysis was performed in *Lemna minor*.

To verify the contribution of organic acid exudation to inorganic Sn tolerance, we examined the stress-alleviating effects of adding 10 μM citrate to 1/2 MS medium containing 50 μM SnCl_4_. The results indicated that 10 μM citrate dramatically attenuated the inhibitory effect of inorganic Sn stress on Arabidopsis and *Lemna minor* root growth (Figure 5). Additionally, as revealed by ICP-MS, citrate supplementation under stress conditions profoundly attenuated Sn accumulation in seedlings, lowering levels from ~28 mg/kg to 9.7 mg/kg, thereby effectively blocking Sn uptake (Figure 5C). Similarly, we treated *Lemna minor* with exogenous citrate, and the results were largely consistent with those in Arabidopsis (Figure 5). Exogenous citrate substantially alleviated the root growth inhibition induced by inorganic Sn stress, and significantly reduced the uptake of inorganic Sn by *Lemna minor*. These results collectively indicate that exogenous citrate can mitigate the inhibitory effects of inorganic Sn stress on plant growth.

### 2.6. Citrate Mitigates Sn Stress-Induced ROS Accumulation

**Figure 6:**
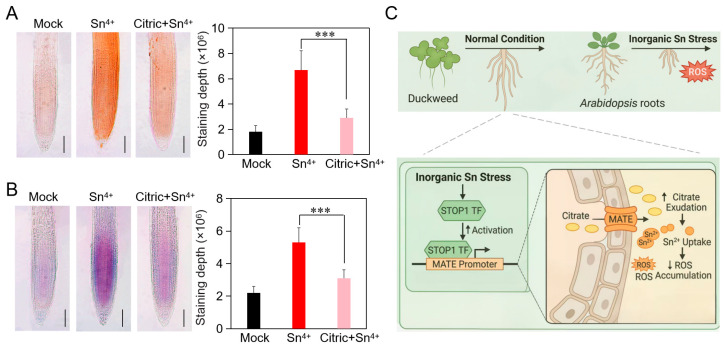
Citrate alleviates Sn-induced ROS accumulation. (**A**) DAB staining of H_2_O_2_ and (**B**) NBT staining of O_2_^−^ in Arabidopsis roots treated with 50 µM SnCl_4_ and/or 10 µM citrate for 1 h. Data are presented as the mean ± SE (*n* > 5). Asterisks indicate statistically significant differences (***, *p* < 0.01). (**C**) Schematic model of the STOP1-MATE-citrate axis conferring inorganic Sn tolerance in plants.

Adverse environmental stresses often lead to the aberrant generation and excessive accumulation of ROS in plants. Unsurprisingly, inorganic Sn stress resulted in the abnormal accumulation of ROS in *Lemna minor* roots. To investigate whether the alleviation of inorganic Sn stress by citrate operates through ROS scavenging, we conducted an analysis using Arabidopsis. DAB and NBT staining further demonstrated that citrate supplementation significantly attenuated inorganic Sn-induced ROS accumulation, which is crucial for mitigating seedling stress (Figure 6A,B). Collectively, these findings clearly establish that exogenous citrate effectively alleviates inorganic Sn stress in plants, largely by impeding Sn absorption and reducing excessive internal ROS accumulation (Figure 6C).

## 3. Discussion

Heavy metal (HM) toxicity represents a major constraint on plant growth and agricultural sustainability. By inducing ionic imbalance, oxidative stress, and metabolic disruption, HMs severely impair plant physiology [10]. While certain HMs act as essential micronutrients at low concentrations but become phytotoxic in excess, others like Pb, Cd, Hg, and As are strictly non-essential and highly deleterious [12,13]. Despite the well-characterized toxicity of these metals, the ecotoxicology of inorganic Sn remains largely overlooked, with its phytotoxicity historically perceived as low [1]. Here, we challenge this notion. Using the model plant Arabidopsis and the environmentally sensitive aquatic *Lemna minor*, we demonstrate that inorganic Sn exerts significant toxicity at remarkably low thresholds; specifically, 25 μM SnCl_4_ impaired the growth of both species, and 50 μM SnCl_4_ drastically inhibited primary root elongation. Given the escalating release of inorganic Sn into ecosystems via industrialization, our findings highlight an underestimated and severe threat to plant health that demands urgent attention.

Root exudation of organic anions is a classic adaptive strategy to mitigate cation toxicity and nutrient deficiency [14]. By chelating toxic cations in the rhizosphere, these ligands reduce their bioavailability to roots [15,16]. Notably, the tricarboxylate citrate exhibits significantly stronger cation-chelating capacity than dicarboxylates (e.g., malate) [17,18]. Given this chemical property, we investigated whether citrate exudation serves as a defense against inorganic Sn stress. Here, we first demonstrated that STOP1, the master regulator of aluminum tolerance, is remarkably sensitive to inorganic Sn. Subsequently, we found that Sn strongly induces the expression of the STOP1-targeted citrate transporter *MATE*, and the *mate* mutant is correspondingly hypersensitive. Al toxicity enhances STOP1 protein stability rather than inducing *STOP1* gene expression [19,20]. Likewise, Sn stress does not increase *STOP1* transcription but significantly upregulates downstream *MATE* expression. Thus, we hypothesize that Sn stress may elicit this response via enhanced STOP1 protein stability, a regulatory mechanism that requires further investigation. Notably, exogenous citrate profoundly rescued Sn-induced root growth inhibition, aligning with its superior chelating capacity. Collectively, these findings establish that plants proactively counteract environmental inorganic Sn stress through the STOP1-MATE-mediated regulation of citrate exudation.

In Arabidopsis, ALMT1-mediated malate exudation plays a dominant role in aluminum (Al) resistance, whereas MATE-mediated citrate exudation contributes to a lesser extent [21,22]. Interestingly, different plant species have evolved distinct organic acid profiles to detoxify Al [23,24,25,26]. However, how plants defend against inorganic Sn toxicity has remained entirely unknown. Here, we reveal a striking mechanistic shift: Arabidopsis primarily defends against inorganic Sn stress by modulating MATE-mediated citrate exudation. This stands in stark contrast to its malate-dependent strategy against Al toxicity. This finding highlights a sophisticated specificity in plant stress responses—rather than employing a universal detoxification mechanism, plants dynamically deploy distinct organic acid exudation profiles to neutralize specific metal ions, a tailored adaptability of paramount importance for surviving heterogeneous environments.

## 4. Materials and Methods

### 4.1. Plant Materials and Growth Conditions

*Arabidopsis thaliana* ecotype Columbia-0 (Col-0) and relevant mutant lines (*stop1*, SALK_114108C; *mate*, SALK_081671; *almt1*, SALK_009629C) obtained from the Nottingham Arabidopsis Stock Centre (NASC, Loughborough, UK) were used. After surface sterilization, seeds of Arabidopsis were sown on 1/2 MS medium containing 1% (*w*/*v*) sucrose, 0.6% (*w*/*v*) agar, and 50 μM SnCl_4_. The plates were placed vertically in a controlled chamber at 22 ± 1 °C under a 16/8 h light/dark cycle with a light intensity of 80–90 µmol m^−2^ s^−1^. Root lengths were measured for statistical analysis on the seventh day after germination. *Lemna minor* was cultured in Hoagland’s solution at 25 °C under a 16/8 h light/dark cycle. Under these conditions, *Lemna minor* reproduces asexually approximately every 48 h. Mature plants of *Lemna minor* exhibiting stable root length and frond size were selected and exposed to 50 μM SnCl_4_. Seven days post-treatment, the root length and frond size of the mature offspring were measured. To detect ROS accumulation, normally grown *Lemna minor* was transferred to fresh Hoagland’s solution with 50 μM SnCl_4_ for 1 h, while vertically grown Arabidopsis seedlings on 1/2 MS medium were transferred to fresh medium containing 50 μM SnCl_4_ for 1 h.

### 4.2. Cytological Observation and Tissue Staining

To detect ROS, *Lemna minor* and Arabidopsis seedlings were stained with 0.1% DAB (3,3′-diaminobenzidine in 50 mM Tris-HCl, pH 5.0) or 0.1% NBT (nitroblue tetrazolium in phosphate buffer, pH 7.0) for up to 30 min, adjusting the incubation time to achieve optimal staining intensity. Cell death was assessed by staining with 0.4% (*w*/*v*) Trypan Blue in a 1:1:2 (*v*/*v*) mixture of lactic acid, glycerol, and distilled water. Following either ROS or cell death staining, samples were cleared in 95% ethanol for 1 h, mounted on slides using HCG solution (24 g chloral hydrate, 3 mL glycerol, 9 mL H_2_O), and imaged with a Leica DM2500 microscope (Wetzlar, Germany). Image J software (v1.54r) was used to quantitatively analyze the results of DAB and NBT staining.

### 4.3. Quantitative Real-Time PCR

Total RNA was extracted from 10-day-old Arabidopsis seedlings or roots using the TaKaRa MiniBEST Plant RNA Extraction Kit (TaKaRa, Osaka, Japan). First-strand cDNA was synthesized using the PrimeScript™ 1st Strand cDNA Synthesis Kit. Quantitative real-time PCR (qRT-PCR) was performed on a QuantStudio 3 system (Thermo Fisher Scientific, Waltham, MA, USA) using SYBR Premix Ex Taq™ II. Unless otherwise specified, all TaKaRa reagents were from Osaka, Japan. Relative transcript levels were calculated using the 2^−ΔΔCt^ method and normalized to the internal reference gene *ACTIN2*. All primer sequences are listed in Table S1.

### 4.4. Determination of Citrate and Malate Content by HPLC-MS/MS

Arabidopsis seedlings grown hydroponically for 7 days were transferred to fresh nutrient solutions supplemented with or without 50 µM SnCl_4_ (control). Following a 3-day culture, the solutions were collected to determine citrate content for assessing citrate efflux. Root exudates were collected by transferring 10-day-old seedlings into sterile 0.5 mM CaCl_2_ solution (pH 5.5) with or without Sn treatment. After a 24 h incubation in the dark, the solutions were filtered (0.22 µm) and lyophilized. The dried residues were reconstituted in 0.1% formic acid for HPLC-MS/MS analysis. Chromatographic separation was performed on a HILIC column, and mass detection was conducted in ESI^−^ mode using MRM. Exudation rates of citrate and malate were quantified via standard curves and expressed as nmol g^−1^ FW h^−1^.

### 4.5. Determination of Sn Content in Plants by ICP-MS

Plant seedlings were washed with 20 mM Na_2_-EDTA and 5 mM CaCl_2_ to remove apoplastic Sn, followed by rinsing with deionized water. Dried samples (0.1 g) were microwave-digested with concentrated HNO_3_ and H_2_O_2_, diluted with ultrapure water, and analyzed for Sn content using ICP-MS (Agilent 7900, Hachioji, Tokyo, Japan) with Indium as the internal standard. Sn concentrations are expressed as mg kg^−1^ DW.

## 5. Conclusions

We demonstrate that the transcription factor STOP1 confers Sn tolerance primarily by activating the MATE-mediated citrate exudation pathway. Mechanistically, secreted citrate detoxifies Sn by restricting its uptake and alleviating ROS overaccumulation. Notably, this STOP1-MATE-citrate axis contrasts sharply with the STOP1-ALMT1-malate axis required for Al tolerance, underscoring that plants deploy distinct organic acid secretion strategies to combat different heavy metal stresses.

## Data Availability

The original contributions presented in this study are included in the article. Further inquiries can be directed to the corresponding authors.

## Supplementary Materials

The following supporting information can be downloaded at: https://www.mdpi.com/article/10.3390/plants15142231/s1, Table S1: Primers used in the study.

## Abbreviations

The following abbreviations are used in this manuscript:

HM	Heavy metal
qPCR	quantitative real-time PCR
STOP1	SENSITIVE TO PROTON RHIZOTOXICITY 1
ALMT1	ALUMINUM-ACTIVATED MALATE TRANSPORTER 1
MATE	MULTI-DRUG AND TOXIC COMPOUND EXTRUSION
DAB	3,3′-diaminobenzidine
NBT	Nitroblue tetrazolium
