# Supplementary material for: The STOP1-MATE-Citrate Axis Confers Inorganic Sn Tolerance in Plants
Source: Plants (Basel). 2026 Jul 22;15(14):2231. doi: 10.3390/plants15142231 (PMC13417497; doi:10.3390/plants15142231)
Supplement: Supplementary file 1 [file plants-15-02231-s001.zip › plants-4411263-supplementary.pdf]

Table S1. Primers used in the study.

Name	Sequences	Process
ALMT1-Q-F	ACTTGAGAGAGCTGAGTGACC	Real-time qPCR
ALMT1-Q-R	TCTTCTCGGGTCTTCATTCCC	Real-time qPCR
MATE-Q-F	GCATAGGACTTCCGTTTGTGGCA	Real-time qPCR
MATE-Q-R	CGAACACAAACGCTAAGGCA	Real-time qPCR
STOP1-Q-F	CCAAGTTCCATCTCAAGCTTTTCT	Real-time qPCR
STOP1-Q-R	TGGGACGTAAAACCTGCGAA	Real-time qPCR
ACT2-Q-F	GCTGACCGTATGAGCAAAGA	Real-time qPCR
ACT2-Q-R	GATCCACATCTGTTGGAACG	Real-time qPCR
